# Nucleolar and spindle associated protein 1 promotes the aggressiveness of astrocytoma by activating the Hedgehog signaling pathway

**DOI:** 10.1186/s13046-017-0597-y

**Published:** 2017-09-12

**Authors:** Xianqiu Wu, Benke Xu, Chao Yang, Wentao Wang, Dequan Zhong, Zhan Zhao, Longshuang He, Yuanjun Hu, Lili Jiang, Jun Li, Libing Song, Wei Zhang

**Affiliations:** 1 State Key Laboratory of Oncology in Southern China and Department of Experimental Research, Sun Yat-sen University Cancer Center, Guangzhou, 510060 China; 2 Department of Anatomy, Medical School of Yangtzeu University, Guangzhou, China; 3 Department of Neurosurgery, First Affiliated Hospital of Sun Yat-sen University, Guangzhou, China; 4 Neurosurgical Research Institute, the First Affiliated Hospital of Guangdong Pharmaceutics University, Guangzhou, 510060 China; 5 Key Laboratory of Protein Modification and Degradation, School of Basic Medical Sciences, Affiliated Cancer Hospital & Institute of Guangzhou Medical University, Guangzhou Medical University, Guangzhou, China; 6 Guangdong Province Key Laboratory of Brain Function and Disease, Department of Biochemistry, Zhongshan School of Medicine, Sun Yat-sen University, Guangzhou, China

**Keywords:** Astrocytoma, NUSAP1, GLI1, Aggressiveness, Hedgehog pathway

## Abstract

**Background:**

The prognosis of human astrocytoma is poor, and the molecular alterations underlying its pathogenesis still needed to be elucidated. Nucleolar and spindle associated protein 1 (NUSAP1) was observed in several types of cancers, but its role in astrocytoma remained unknown.

**Methods:**

The expression of NUSAP1 in astrocytoma cell lines and tissues were measured with western blotting and Real-Time PCR. Two hundred and twenty-one astrocytoma tissue samples were analyzed by immunochemistry to demonstrate the correlation between the NUSAP1 expression and clinicopathological characteristics. 3-(4,5-dimethylthiazol-2-yl) 2,5-diphenyltetrazolium bromide (MTT) assay, colony formation, transwell matrix penetration assay, wound healing assay and anchorage-independent growth assay were used to investigate the biological effect of NUSAP1 in astrocytoma. An intracranial brain xenograft tumor model was used to confirm the oncogenic role of NUSAP1 in human astrocytoma. Luciferase reporter assay was used to investigate the effect of NUSAP1 on Hedgehog signaling pathway.

**Results:**

NUSAP1 was markedly overexpressed in astrocytoma cell lines and tissues compared with normal astrocytes and brain tissues. NUSAP1 was found to be overexpressed in 152 of 221 (68.78%) astrocytoma tissues, and was significantly correlated to poor survival. Further, ectopic expression or knockdown of NUSAP1 significantly promoted or inhibited, respectively, the invasive ability of astrocytoma cells. Moreover, intracranial xenografts of astrocytoma cells engineered to express NUSAP1 were highly invasive compared with the parental cells. With regard to its molecular mechanism, upregulation of NUSAP1 in astrocytoma cells promoted the nuclear translocation of GLI family zinc finger 1 (GLI1) and upregulated the downstream genes of the Hedgehog pathway.

**Conclusion:**

These findings indicate that NUSAP1 contributes to the progression of astrocytoma by enhancing tumor cell invasiveness via activation of the Hedgehog signaling pathway, and that NUSAP1 might be a potential prognostic biomarker as well as a target in astrocytoma.

**Electronic supplementary material:**

The online version of this article (10.1186/s13046-017-0597-y) contains supplementary material, which is available to authorized users.

## Background

Glioma is the most common primary brain tumor in adults, and it is also one of the most fatal human cancers. Despite various improvements in cancer treatment over the last two decades, the outcome of patients with malignant glioma remain very poor [1–3]. It has been reported that the cumulative 1-year survival rate of glioma patient is no more than 30%; moreover, the overall median survival for glioblastoma, the most lethal brain tumor, ranges from 1.4 to 1.8 years, with only a third of patients surviving for 1 year and less than 5% surviving beyond 5 years [4, 5]. One of the reasons why patients with glioma have such a low survival rate is that glioma tumor cells have a high degree of invasiveness [6–8].

The World Health Organization (WHO) classifies gliomas according to the cells that the tumor cells morphologically resemble (astrocytes, oligodendrocytes, or a mixture of both cell types) and groups the tumors into four grades based on histological features and aggressiveness [9]. Astrocytoma, including glioblastoma, which is the most common primary tumor type of human glioma, accounts for 75% of all gliomas [10]. The clinical prognosis of astrocytoma is still mainly dependent on conventional pathological parameters, such as the histological type and tumor grade. Even though the WHO histopathological classification is widely used, it is limited by substantial interobserver variability and poor correlation with the clinical outcomes. The progression of astrocytoma is related to various molecular alterations, but these molecular mechanisms have not been adequately elucidated. Therefore, clarifying the molecular mechanisms of astrocytoma and identifying prognostic factors as well as potential targets would have great clinical value.

Hedgehog (HH), which was first identified in *Drosophila melanogaster* in the 1980s, plays a critical role in early embryonic development [11]. It has three vertebrate homologs that function as ligands: Sonic hedgehog (SHH), Indian hedgehog (IHH) and desert hedgehog (DHH) [12]. Smoothened (SMO), a 7-transmembrane protein related to the G protein-coupled receptor, and glioma-associated oncogene (GLI) are the two major HH signal transducers. There are three GLI proteins in vertebrates: GLI1, GLI2, and GLI3. In brief, HH ligands bind to Patched (PTCH1) and lead to the release of SMO into the primary cilia, which in turn results in dissociation of the suppressor-of-fused (SUFU)-GLI complex. The dissociation of this complex leads to the nuclear translocation and activation of GLI1 and GLI2, as well as the degradation of GLI3 [13]. GLI1 can also reinforce GLI activity via a positive feedback mechanism [14]. Recent evidence has shown that the HH pathway plays an important role in a broad range of tumors, for example, basal cell carcinoma [15, 16], small cell lung cancer [17], prostate cancer [18], gastric cancer [19], esophageal cancer [20], pancreatic cancer [19, 21], and hepatocellular carcinoma [22, 23]. It was reported that up to 30% of medulloblastomas (a primitive neuroectodermal tumor) exhibit activation of the HH pathway [24–26]. The HH pathway has been reported to be hyper-activated in multiple human tumors, including gliomas [27–30]. For instance, the expression of PTCH1, the HH receptor, is significant higher in grade II/III gliomas and sonic hedgehog, one of the HH ligands, overexpresses in 80% of the human glioblastoma multiforme (GBM) [27, 28]. Further studies showed that 66.7% primary and recurrent gliomas showed Gli1-nuclear expression, which positively correlated with glioma progression [29, 30]. However, HH pathway driver mutations are thought to be of low frequency in gliomas, suggesting that alternative mechanism is involved in activation of HH pathway in gliomas.

Nucleolar spindle-associated protein 1 (NUSAP1) is a microtubule-associated protein that plays an important role in spindle assembly, chromosome segregation, cytokinesis, and microtubule crosslinking, bundling and attachment to chromosomes [31, 32]. NUSAP1 was identified as a microtubule stabilizer as a result of its ability to induce microtubule crosslinking, bundling, and attachment to chromosomes [33, 34]. High expression of NUSAP1 has been observed in several types of tumors, such as pancreatic adenocarcinoma, acute myeloid leukemia and prostate cancer [35–38]. However, although a number of studies have explored the role of NUSAP1 in various tumors, its role in astrocytoma remains unknown.

In an effort to understand the role of NUSAP1 in astrocytoma, the present study investigates the expression of NUSAP1 in astrocytoma cell lines and tissues. Our findings indicated that NUSAP1 played an important role in promoting aggressiveness in astrocytoma via activating the HH pathway. Thus, NUSAP1 might be a useful prognostic biomarker and a potential target in the diagnosis and treatment of astrocytoma.

## Methods

### Cell lines

Primary normal human astrocytes (NHAs) were purchased from Sciencell Research Laboratories. The glioma cell lines U-118MG, U-87MG, A-172, SW 1088, SW 1788 and LN-18 were purchased from American Type Culture Collection (ATCC). These cells were grown in Dulbecco’s modified Eagle medium (DMEM) supplemented with 10% fetal bovine serum (FBS). U138MG was also purchased from ATCC and cultured in DMEM supplemented with 10% FBS. Fresh brain tumor tissues that were clinically histopathologically diagnosed at the Sun Yat-sen University-Affiliated First Hospital were used. Patient’s consent and approval from the Institutional Research Ethics Committee were acquired for use of data for the research. All the tissues were collected and processed within 30 min after resection. The primary cultured tumor cells were obtained after mechanical dissociation, as previously described [39].

### Patient information and tissue specimens

A total of 221 paraffin-embedded glioma samples, including WHO grade II–IV tumors, were used in this study. All of them were both clinically and pathologically diagnosed at the Sun Yat-sen University-Affiliated First Hospital between 2000 and 2010. The clinicopathological characteristics of the specimens are shown in Additional file 1: Table S1. Normal brain tissues were obtained from individuals who had died from traffic accidents and confirmed to be free of any preexisting pathologically detectable conditions. Prior donor consent and the approval of the Institutional Research Ethics Committee were obtained for use of the data. Clinical and pathological classification was conducted according to the seventh edition of the classification system of the American Joint Committee on Cancer (AJCC).

### RNA extraction, reverse transcription and real-time RT-PCR

Total RNA was extracted from cell lines and freshly frozen samples with TRIzol reagent (Invitrogen, Carlsbad, CA, USA) and was reverse-transcribed with the first-strand cDNA synthesis kit (Invitrogen). Real-time PCR reactions were conducted using Platinum SYBR Green qPCR SuperMix-UDG reagents (Invitrogen). Reverse transcriptase was used as the negative control, and glyceraldehyde-3-phosphate dehydrogenase (GAPDH) was used as the endogenous control. All experiments were repeated twice. The 2^-ΔΔCT^ equation was used to calculate the relative expression levels. The PCR primers used in this study were as follows: NUSAP1 (5′: GAAGCGCGGCATTCTTCATT, 3′: CGGCGATACTCGGAAGATGG), GAPDH (5′: CACCATCTTCCAGGAGCGAG, 3′: GACTCCACGACGTACTCAGC), GLI1 (5′: GCTCTGGACATACCCCACCT,3′: GCAGCTCCCCCAATTTTTCTG), PTCH1 (5′: TCGCTCTGGAGCAGATTTCC, 3′: TCTCGAGGTTCGCTGCTTTT), HIP1 (5′: GGCGACATGGATCGGATGG, 3′: ACAGCCACTTCCTGCGTATT), CCND1 (5′: AAAGAATTTGCACCCCGCTG, 3′: GACAGACAAAGCGTCCCTCA), CCNE1 (5′, GCAGGATCCAGATGAAGAAATG, 3′: TAATCCGAGGCTTGCACGTT), HDAC1 (5′: TGCAAAGAAGTCCGAGGCAT, 3′: ACCCTCTGGTGATACTTTAGCA).

### Vectors, retroviral infection and transfection

PMSCV/NUSAP1 was generated by subcloning the PCR-amplified human NUSAP1 coding sequence into the pMSCV vector (Clontech). Human NUSAP1 targeting shRNA oligonucleotide sequences (RNA#1: 5-GCACCAAGAAGCTGAGAATGC-3, and RNA#2: 5′-GGAAATGGAGTCCATTGATCA-3) were cloned to generate pSuperretro-NUSAP1-shRNA(s). The Lipofectamine 3000 reagent (Invitrogen) was used for transfecting plasmids. Retroviral production and infection were performed as described previously [40]. Stable cell lines expressing NUSAP1 or NUSAP1 shRNA were selected for 10 days by treatment with 0.5 μg/ml puromycin for 48 h after infection. In the same way, stable cell lines expressing Nusap1 shRNA (RNAi#1, 5-GCATGTTAAGGAAACTCAGCC-3, and RNAi#2, 5-GCAGCGCCTCATCAAGAAAGT-3) were established and selected. Human GLI1 targeting shRNA oligonucleotides sequences were as follows: RNA#1: 5-GCCACCAAGCTAACCTCATGT-3, and RNA#2: 5-GCCTGAATCTGTGTATGAAAC-3.

### Western blot analysis

Western blot analysis was conducted using anti-NUSAP1, anti-GAPDH, anti-α-tubulin, anti-MMP2, anti-MMP9, anti-Ki67, anti-β-actin, and anti-elongation factor 1 alpha (EF1α) antibodies (Abcam, Cambridge, MA, USA). Human GAPDH, α-tubulin, β-actin, or EF1-α were used as the endogenous reference.

### Immunohistochemistry

Immunohistochemistry was performed in 221 clinical glioma tissue sections using a previously described method [41]. The degree of immunostaining was reviewed and scored separately by two independent pathologists blindly. The scores were determined by combining the proportion of positively-stained tumor or normal pancreatic epithelial cells and the intensity of staining. Cell proportions were scored as follows: 0, no positive cells; 1, < 10% positive cells; 2, 10%–35% positive cells; 3, 35%–75% positive cells; 4, > 75% positive cells. Staining intensity was graded according to the following standard: 1, no staining; 2, weak staining (light yellow); 3, moderate staining (yellow brown); 4, strong staining (brown). The staining index (SI) was calculated as the product of the staining intensity score and the proportion of positive cells. Using this method of assessment, we evaluated protein expression of NUSAP1 in glioma specimens by determining the SI, with possible scores of 0, 2, 3, 4, 6, 8, 9, 12, and 16. Sample with a score index ≥ 8 were determined as high expression and samples with a score index < 8 were determined as low expression.

### MTT assay

Cells (5 × 10^3^ per well) were seeded in 96-well culture plates and stained with 100 μl of sterile MTT dye (0.5 mg/ml; Sigma, St. Louis, Missouri, USA) at 1, 2, 3, 4 and 5 days; this was followed by additional incubation for 4 h at 37 °C. After removal of the culture medium from each well, 150 μl of dimethyl sulfoxide (Sigma, St. Louis, MO, USA) was added and thoroughly mixed for 15 min. Following this, a microplate reader (Bio-Rad 3500; Hercules, California, USA) was used to determine the optical density, and absorbance was measured at a wavelength of 570 nm with a reference wavelength of 655 nm. All the experiments were repeated three times.

### Colony formation

The indicated cells were plated in 6-well plates (1 × 10^3^ cells per well) and cultured for 2 weeks. The colonies were fixed with methanol for 10 min and stained with 1% crystal violet for 1 min. All the experiments were repeated three times.

### Transwell migration assay and Transwell matrix penetration assay

For the Transwell assay or Transwell matrix penetration assay, the indicated cells (1 × 10^4^) were plated on the upper side of a polycarbonate Transwell filter with or without Matrigel in the upper chamber of the BioCoat™ invasion chambers (BD, Bedford, MA). After 22 h of incubation at 37 °C, the cells in the upper chamber were removed with cotton swabs, and the migrated and invaded cells on the lower membrane surface were fixed in 1% paraformaldehyde and stained with hematoxylin. The cells were counted (ten random 100× fields per well) and expressed as the mean number of cells per field of view. All the experiments were repeated three times, and the data were expressed as mean ± standard deviation (SD) values.

### Wound healing assay

The indicated cells were cultured on 6-well plates with DMEM containing 10% FBS. Until the cells become confluence, we made a 500-μm wide cell-free gap by scratching the bottom of the plate with a pipette tip, and the cells were further incubated for 24 h. Phase-contrast images of the wound healing process were obtained digitally using an inverted Olympus IX50 microscope with a 10× objective lens at 0 and 24 h after the scratching. Then, the length of the healed wound was compared with the length of the initial wound.

### Anchorage-independent growth assay

The indicated cells were trypsinized and suspended in complete medium containing 0.3% agar. The cell-agar mixture was plated on the top of a bottom layer with 1% agar-containing medium. About 10 days later, viable colonies that were larger than 0.5 mm in diameter were counted. All the experiments were repeated three times.

### Luciferase assay

The indicated cells were co-transfected with the indicated plasmids and luciferase reporter plasmids in 6-well plates and culture for 48 h, after which the cells were harvested and lysed for luminescence detection. The procedure and detection were performed with a luciferase assay kit according to the manufacturer’s protocol. Renilla luciferase was activated for emission of primary luminescence. All the experiments were repeated three times.

### Intracranial brain tumor xenografts, immunohistochemistry, and hematoxylin-eosin staining

The animal studies were approved by the Ethics Committee of Sun Yat-Sen University, and all the experiments conform to the relevant regulatory standards. SW 1088 cells (5 × 10^5^), SW 1088/NUSAP1 cells (5 × 10^5^), U-87 MG/Scramble cells (5 × 10^5^), or U-87 MG/NUSAP1 shRNA#1 cells (5 × 10^5^) were stereotactically implanted into the brain of nude mice (five mice per group). The tumor-bearing mice were sacrificed 5 weeks after implantation, and the whole brain was resected. The brain specimens were cut to about 4-μm sections and embedded in paraffin for immunohistochemistry and hematoxylin-eosin (H&E) staining. After deparaffinization, immunohistochemistry was conducted using an anti-NUSAP1 antibody. Deparaffinized tumor sections were stained with Mayer’s hematoxylin solution for H&E staining. Images were captured using the AxioVision Rel.4.6 computerized image analysis system (Carl Zeiss).

### Statistical analysis

All statistical analyses were carried out with SPSS v13.0 (SPSS Inc., Chicago, IL, USA). The relationship between NUSAP1 expression and clinicopathological characteristics was analyzed by the chi-square test. Bivariate correlations between study variables were determined using Spearman’s rank correlation coefficients. Survival curves were plotted using the Kaplan-Meier method and the log-rank test. Survival data were evaluated using univariate and multivariate Cox regression analyses. *P* values less than 0.05 were considered to indicate statistical significance.

## Results

### Upregulation of NUSAP1 in astrocytoma cell lines and tissues

**Fig. 1:**
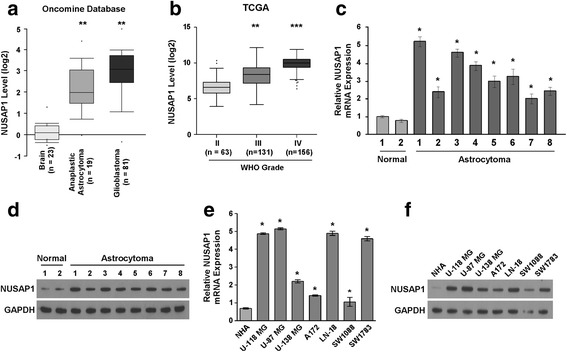
Upregulation of NUSAP1 expression in astrocytoma cell lines and tissues. **a** Based on data obtained from the Oncomine database, the expression of NUSAP1 was markedly upregulated in anaplastic astrocytoma (*n* = 19) and glioblastoma (*n* = 81) compared with normal brain tissues (*n* = 23). Error bars represent the mean ± SD values (**P < 0.05). **b** NUSAP1 expression was upregulated in grade IV astrocytoma (*n* = 156), followed by grade III astrocytoma (*n* = 131) and grade II astrocytoma (*n* = 63), according to The Cancer Genome Atlas (TCGA) database. Error bars represent the mean values determined using the Tukey test (**P < 0.05). **c** and **d** Real-time RT-PCR and western blotting analysis of NUSAP1 expression in astrocytoma tissue and brain tissue. The average NUSAP1 mRNA expression was normalized to the expression of GAPDH. GAPDH was used as a loading control. Error bars represented the mean ± SD values of three independent experiments (*P < 0.05). **e** and **f** Expression of NUSAP1 was upregulated in all seven astrocytoma cell lines compared with primary normal human astrocytes (NHAs), as confirmed by RT-PCR and western blotting analyses. GAPDH was used as a loading control. Error bars represent the mean ± SD values of three independent experiments (*P < 0.05)

We first analyzed the expression levels of NUSAP1 in astrocytoma, including anaplastic astrocytoma (*n* = 19) and glioblastoma (*n* = 81), using data deposited in the Oncomine database. NUSAP1 expression was found to be upregulated in astrocytoma compared to normal brain tissues (Fig. 1a). Further, analysis of NUSAP1 expression from data deposited in The Cancer Genome Atlas (TCGA) database revealed that the increase in the mRNA expression of NUSAP1 in astrocytoma tissues was in tandem with the increase in the WHO grade of the tumor (Fig. 1b). To further confirm these results obtained from public datasets, we examined the expression of NUSAP1 in two normal brain tissues, eight astrocytoma tissues, one primary normal human astrocyte (NHA) cell line and seven astrocytoma cell lines by real-time PCR and western blotting. Real-time PCR and western blotting analyses revealed that the mRNA and protein expression of NUSAP1 was higher in the eight astrocytoma tissues than in the two normal brain tissues (Fig. 1c and d). Compared with the NHA cell line, the expression of NUSAP1 was also upregulated in all seven astrocytoma cell lines, as confirmed by RT-PCR analyses and western blotting (Fig. 1e and f). Thus, our data indicated that the expression of NUSAP1 was upregulated in astrocytoma cell lines and tissues.

### NUSAP1 overexpression correlated with poor survival in patients with astrocytoma

**Fig. 2:**
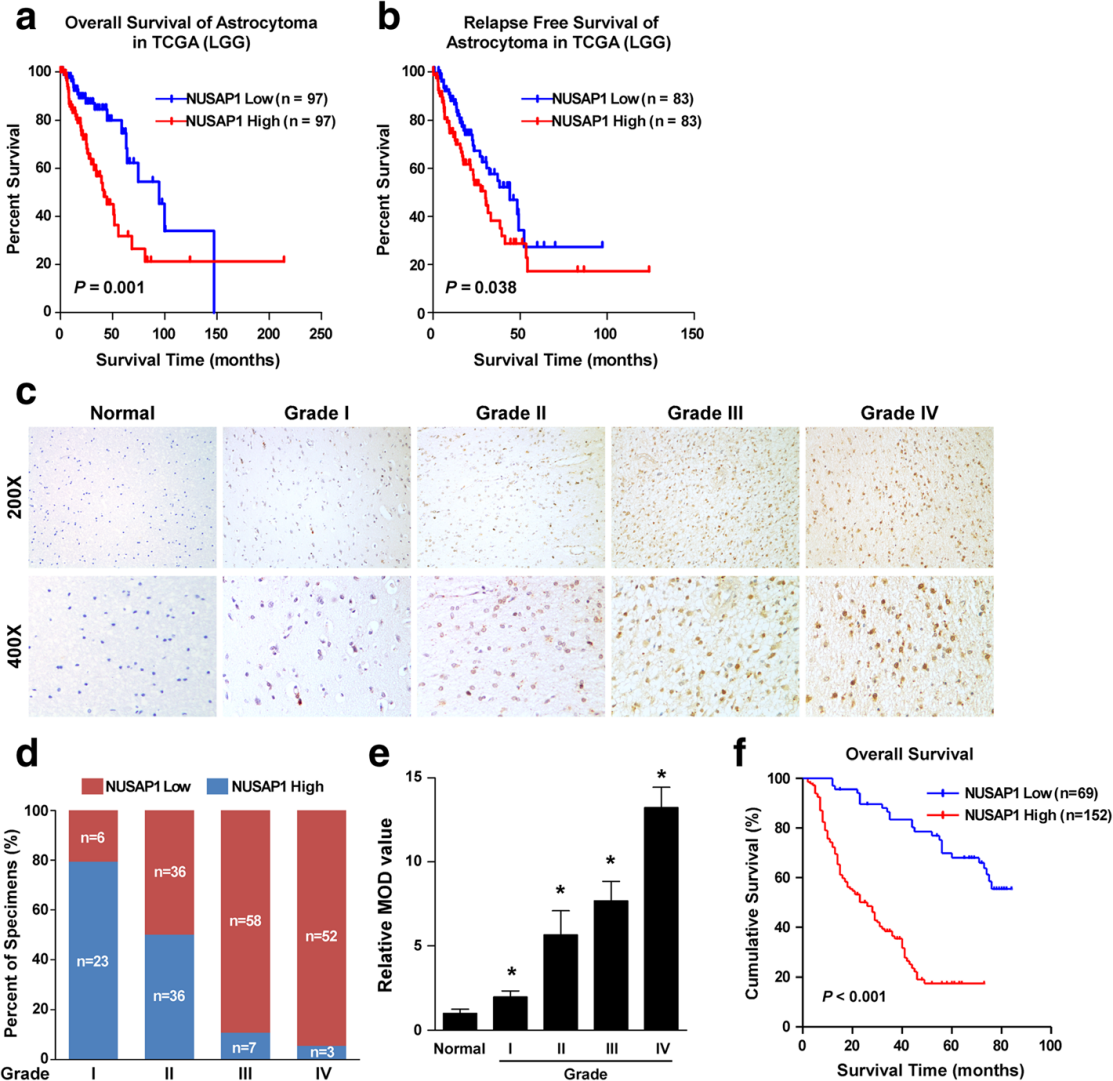
Association of NUSAP1 upregulation with poor survival in astrocytoma. (A and B) Kaplan-Meier overall survival and relapse-free survival curves for patients with astrocytoma (selected from LGG data of TCGA database) stratified by high and low expression of NUSAP1. **c** Representative images of NUSAP1 expression in astrocytoma of different grade. **d** Immunohistochemical analysis of astrocytoma tissue samples of different grades according to the level of NUSAP1 expression. **e** Statistical quantification of the average MODs of NUSAP1 staining of glioma specimens with different WHO grades. **f** Kaplan-Meier overall survival curves for patients with astrocytoma stratified by low (*n* = 69) and high (*n* = 152) expression of NUSAP1 (*P* < 0.001). *, *P* < 0.05)

As the expression of NUSAP1 was upregulated in astrocytoma, we further investigated whether there was a correlation between NUSAP1 expression and survival. We analyzed the expression of NUSAP1 in astrocytoma samples which were selected from low grade glioma (LGG) data of the TCGA databases to perform the survival analysis and found that astrocytoma patients with high NUSAP1 expression had poorer overall survival than patients with low NUSAP1 expression (Fig. 2a). Moreover, upregulation of NUSAP1 was closely related to shorter relapse-free survival (Fig. 2b). To investigate the association between NUSAP1 and clinical characteristics of the patients, we examined NUSAP1 expression by immunohistochemical analysis of 221 paraffin-embedded astrocytoma tissues (Additional file 1: Table S1). Among the NUSAP1-positive cases, 69 (31.22%) had low NUSAP1 expression, while 152 (68.78%) had high NUSAP1 expression (Additional file 1: Table S1). Furthermore, NUSAP1 expression increased markedly with human glioma WHO grade and quantitative IHC analysis revealed that the mean optical density (MOD) of NUSAP1 staining in glioma cells increased significantly with the WHO grade, consistently (Fig. 2, C-E), suggesting that high NUSAP1 protein expression contributes to glioma progression.

Moreover, the statistical analysis indicated that upregulated NUSAP1 protein was associated with WHO tumor grade (*P* < 0.001) and vital status (*P* < 0.001) (Additional file 1: Table S2). Kaplan–Meier analysis and log-rank testing revealed that NUSAP1 expression levels were inversely correlated with survival time, whether at WHO grade I–II or at WHO grade III–IV (Fig. 2F; Additional file 2: Figure S1). Moreover, univariate and multivariate survival analyses revealed that NUSAP1 expression was an independent prognostic factor of glioma (*P* < 0.001) similar to the WHO grade (*P* < 0.001) (Additional file 1: Table S3). Taken together, NUSAP1 protein upregulation in glioma contributes to glioma progression and correlates with poor prognosis of the disease.

### Upregulation of NUSAP1 augmented the aggressiveness of astrocytoma in vitro

**Fig. 3:**
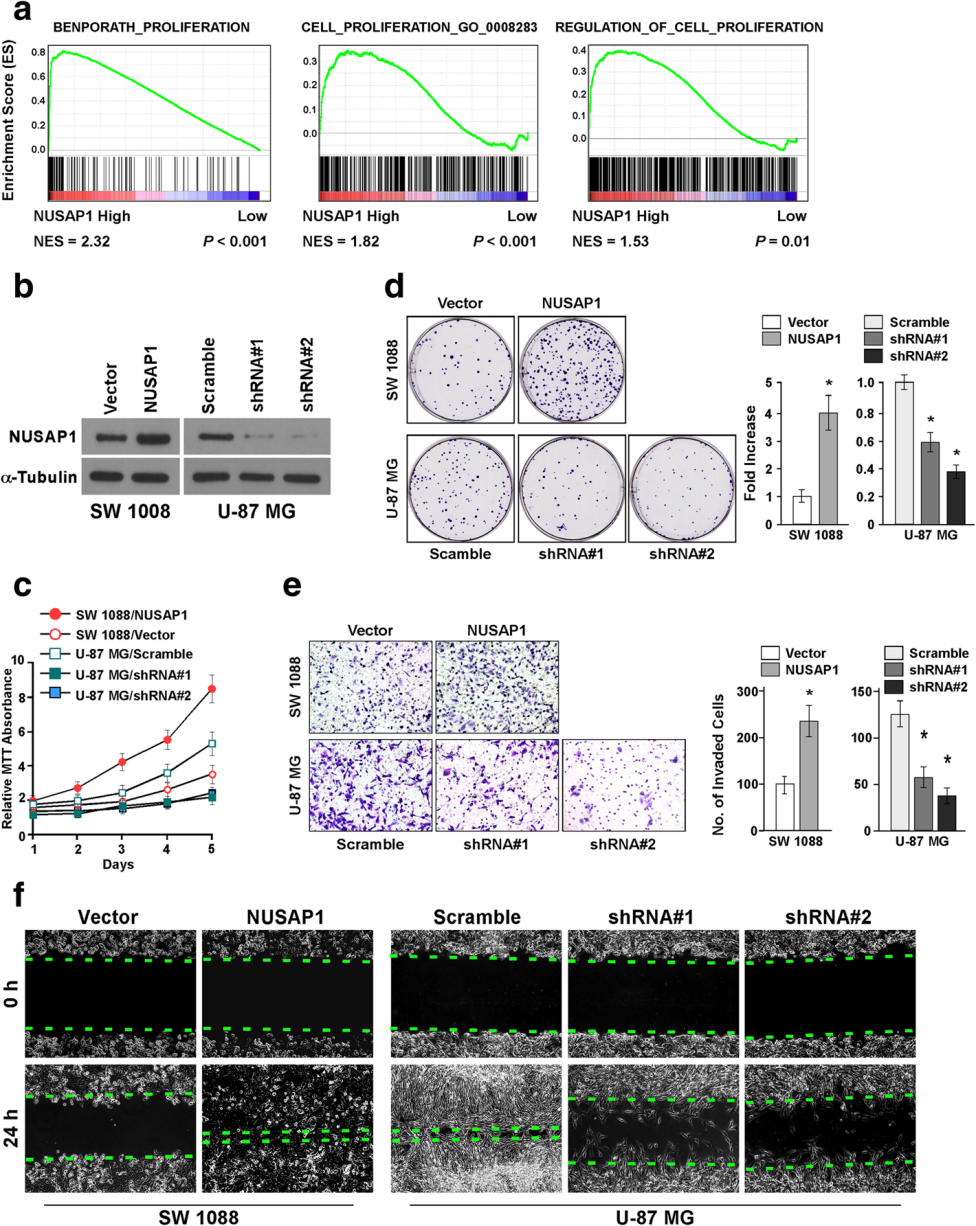
In vitro promotion of aggressiveness in astrocytoma by the upregulation of NUSAP1. **a** Gene set enrichment analysis (GSEA) showed a significant correlation between NUSAP1 mRNA expression and the proliferation-associated gene signature. **b** Western blotting was used to examine the protein expression of NUSAP1 in SW 1008 and U-87 MG cells. α-Tubulin was used as a loading control. **c** In the MTT assay, overexpression of NUSAP1 significantly increased the growth rate of the indicated cells, while downregulation of NUSAP1 decreased the growth rate of the indicated cells. Error bars represent the mean ± SD values of three independent experiments. **d** Representative images (left panel) and quantification (right panel) of cells in the colony formation assay. Overexpression of NUSAP1 increased, while downregulation of NUSAP1 decreased, the colony-forming ability of the indicated cells. Error bars represent the mean ± SD values of three independent experiments (*P < 0.05). **e** Representative images (left panel) and quantification (right panel) of the indicated invaded cells analyzed by the Transwell matrix penetration assay. Error bars represent the mean ± SD values of three independent experiments (*P < 0.05). **f** The wound-healing assay was conducted with the indicated cells, and images were taken at 0 and 24 h. Overexpression of NUSAP1 increased, while downregulation of NUSAP1 decreased, the migration ability of the indicated cells

To investigate whether NUSAP1 has an effect on the pathogenesis of astrocytoma, we used gene set enrichment analysis (GSEA) to predict the possible biological functions of NUSAP1 in the cancer. The findings indicated that NUSAP1 might be involved in proliferation of the cancer cells (Fig. 3a). To verify this, several stable cell lines were established: the SW 1008 and A172 cells stably expressed ectopic NUSAP1, while NUSAP1 expression was silenced in the U-87 MG and A172 cells by small hairpin RNA (shRNA) (NUSAP1 expression was confirmed in these cell lines by western blotting) (Fig. 3b and Additional file 3: Figure S2A). The effect of NUSAP1 on proliferation of astrocytoma cells was evaluated by overexpression and silencing of NUSAP1 transcripts. The 3-(4, 5-dimethyl-2-thiazolyl)-2,5-diphenyl-2H-tetrazolium bromide (MTT) assay showed that the NUSAP1-transduced astrocytoma cells (SW 1088 and A172 cells) displayed a significant increase in viability, while the NUSAP1-silenced U-87 MG and A172 cells displayed contrasting results (Fig. 3c and Additional file 3: Figure S2B). These results were further confirmed by the colony formation assay (Fig. 3d and Additional file 3: Figure S2C). Furthermore, in the Transwell matrix penetration assay, overexpression of NUSAP1 was found to enhance invasion in the SW 1088 and A172 cells, while knockdown of NUSAP1 inhibited cell invasion in the U-87 MG and A172 cells (Fig. 3e and Additional file 3: Figure S2D). We also found that the NUSAP1-transduced astrocytoma cells displayed an increase in migration ability, while the NUSAP1-silenced cells displayed a decrease in their migration ability in the wound healing assay (Fig. 3f and Additional file 3: Figure S2E). These observations indicated that upregulation of NUSAP1 promoted the proliferation, invasion and migration of astrocytoma cells.

### Upregulation of NUSAP1 promotes the aggressiveness of astrocytoma in vivo

**Fig. 4:**
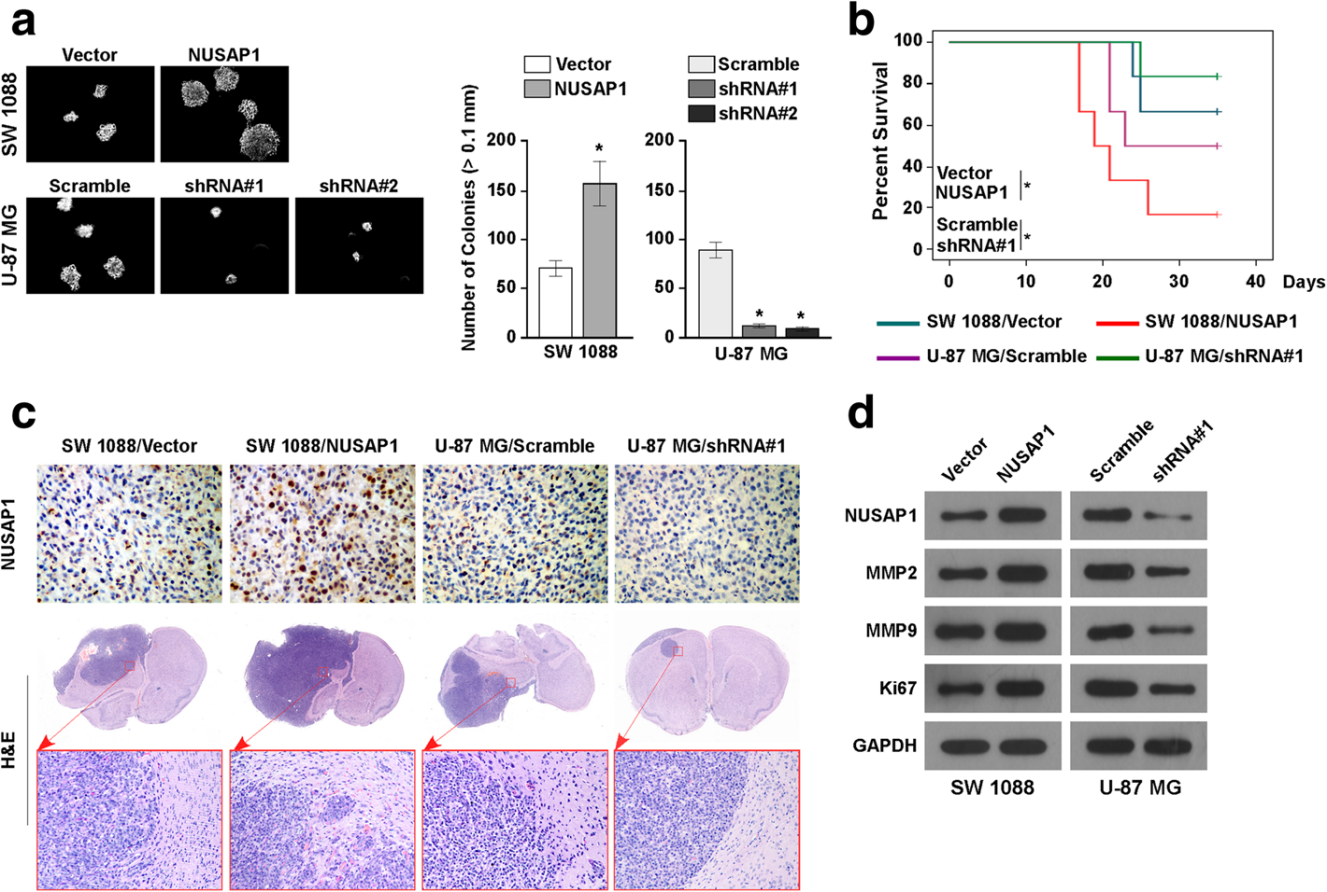
In vivo promotion of the aggressiveness of astrocytomas by overexpression of NUSAP1. **a** Representative images (left panel) and quantification (right panel) of the indicated invaded cells by the anchorage-independent growth assay. Error bars represent the mean ± SD values of three independent experiments (*P < 0.05). **b** Survival curves of mice with brain astrocytoma xenografts formed by the indicated cells (*P < 0.05). **c** Representative images of the tumor formed from the indicated cells in the intracranial brain tumor xenograft nude mice. Immunohistochemistry staining (upper panel) with the NUSAP1 antibody and hematoxylin-eosin staining (lower panel) demonstrated that overexpression of NUSAP1 induced, whereas downregulation of NUSAP1 inhibited, the aggressive phenotype of astrocytoma cells in vivo. **d** Western blotting was used to assess the expression of NUSAP1, MMP2, MMP9 and Ki67 in the indicated cells. GAPDH was used as a loading control

As indicated by the above results, NUSAP1 promoted the aggressiveness of astrocytoma. Therefore, we tried to confirm whether NUSAP1 had the same effect in vivo. Before conducting the in vivo experiment, we simulated the internal environment to evaluate the growth ability of astrocytoma cells via an anchorage-independent growth assay. As demonstrated by the anchorage-independent growth assay, overexpression of NUSAP1 markedly increased the anchorage-independent growth ability of SW 1088 and A172 cells, while suppression of NUSAP1 reduced that growth ability of U-87 MG and A172 cells (Fig. 4a and Additional file 3: Figure S2F). Subsequently, the SW 1088 and U-87 MG cell lines, which stably overexpressed and suppressed NUSAP1 expression, respectively, were stereotactically implanted into the brain of nude mice; there was also a control group in which a control astrocytoma cell line was implanted. Upregulation of NUSAP1 significant shortened the survival of mice, while silencing of NUSAP1 increased the survival of mice (Fig. 4b). Immunohistochemical staining with an anti-NUSAP1 antibody showed that there was a significant increase in the positive staining densities of NUSAP1-overexpressing tumor samples as compared to the control tumor samples, while the positive staining density was decreased significantly in NUSAP1-suppressed samples (Fig. 4c). The findings also consistently showed that the tumor margins of the NUSAP1-overexpressing tumor samples were more incomplete, while the tumor margins of NUSAP1-suppressed tumor specimens were more complete. Thus, the findings indicate that NUSAP1 promotes aggressiveness in astrocytoma in vivo. To understand the underlying mechanism, we examined whether NUSAP1 could alter the extracellular matrix around the tumor cells. As revealed by western blotting, MMP2, MMP9 and Ki67 showed strong expression in the NUSAP1-transduced cell line, while MMP2, MMP9 and Ki67 expression was weak in the NUSAP1-knockdown cell lines (Fig. 4d and Additional file 3: Figure S2G). Collectively, these data indicated that overexpression of NUSAP1 promoted aggressiveness in astrocytoma.

### Overexpression of NUSAP1 activates HH signaling

**Fig. 5:**
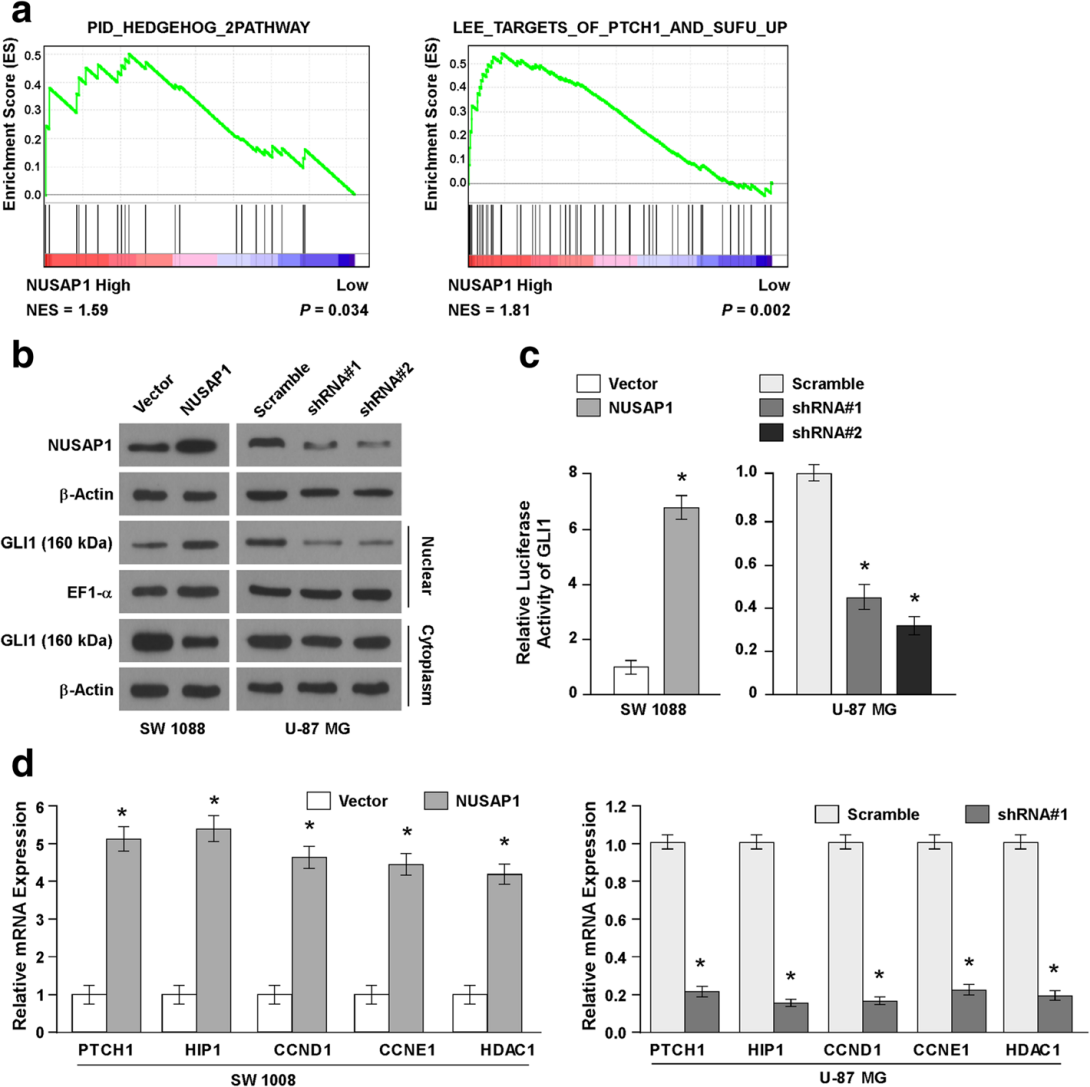
Activation of Hedgehog signaling by overexpression of NUSAP1. **a** Gene set enrichment analysis (GSEA) showed that there was a significant correlation between NUSAP1 mRNA expression and the Hedgehog pathway gene signature. **b** Western blotting was used to examine the expression of NUSAP1 and GLI1 in the indicated cells. β-actin and EF1-α were used as loading controls. **c** Relative activity of reporter luciferase linked to NUSAP1 and GLI1 in the indicated cells. Error bars represent the mean ± SD values of three independent experiments (*P < 0.05). **d** real-time-PCR detection of PTCH1, HIP1, CCND1, CCNE1 and HDAC1 gene expression in SW 1008 and U-87 MG cells. Error bars represent the mean ± SD values of three independent experiments (*P < 0.05)

Previously, Peterson KA et al. defined a prioritized set of 841 enriched Gli1-binding regions (GBRs), including NUSAP1 [42] which prompted us guess to whether there was any association between HH pathway and NUSAP1. Interestingly, GSEA analysis revealed that the expression level of NUSAP1 in TCGA astrocytoma dataset was positively correlated with the HH pathway gene signature (Fig. 5a), further suggested that NUSAP1 may play a role in activation of HH pathway. Furthermore, we found that upregulation of NUSAP1 resulted in an increase in the protein level of GLI1 in the nucleus, while the protein level of GLI1 in cytoplasm was decreased (Fig. 5b and Additional file 4: Figure S3A). Further, downregulation of NUSAP1 could decrease the protein level of GLI1 in the nucleus, while slightly altering GLI1 expression in the cytoplasm. Consistent with the results of western blotting, the Luciferase reporter assays showed that the activity of GLI1 was upregulated in NUSAP1-transduced cells and downregulated in NUSAP1-knockdown cells (Fig. 5c and Additional file 4: Figure S3B). Subsequently, we detected several target genes downstream of the HH signaling pathway by real-time PCR, including PTCH1, HIP1, CCND1, CCNE1 and HDAC1 (Fig. 5d and Additional file 4: Figure S3C-D). Upregulation of NUSAP1 increased, while downregulation NUSAP1 decreased, the expression of PTCH1, HIP1, CCND1, CCNE1 and HDAC1. Our data indicated that NUSAP1 promoted GLI1 translocation to the nucleus from the cytoplasm and subsequently led to activation of the HH signaling pathway in astrocytoma cells.

### Promotion of tumor aggressiveness by overexpression of NUSAP1 via activation of HH signaling

**Fig. 6:**
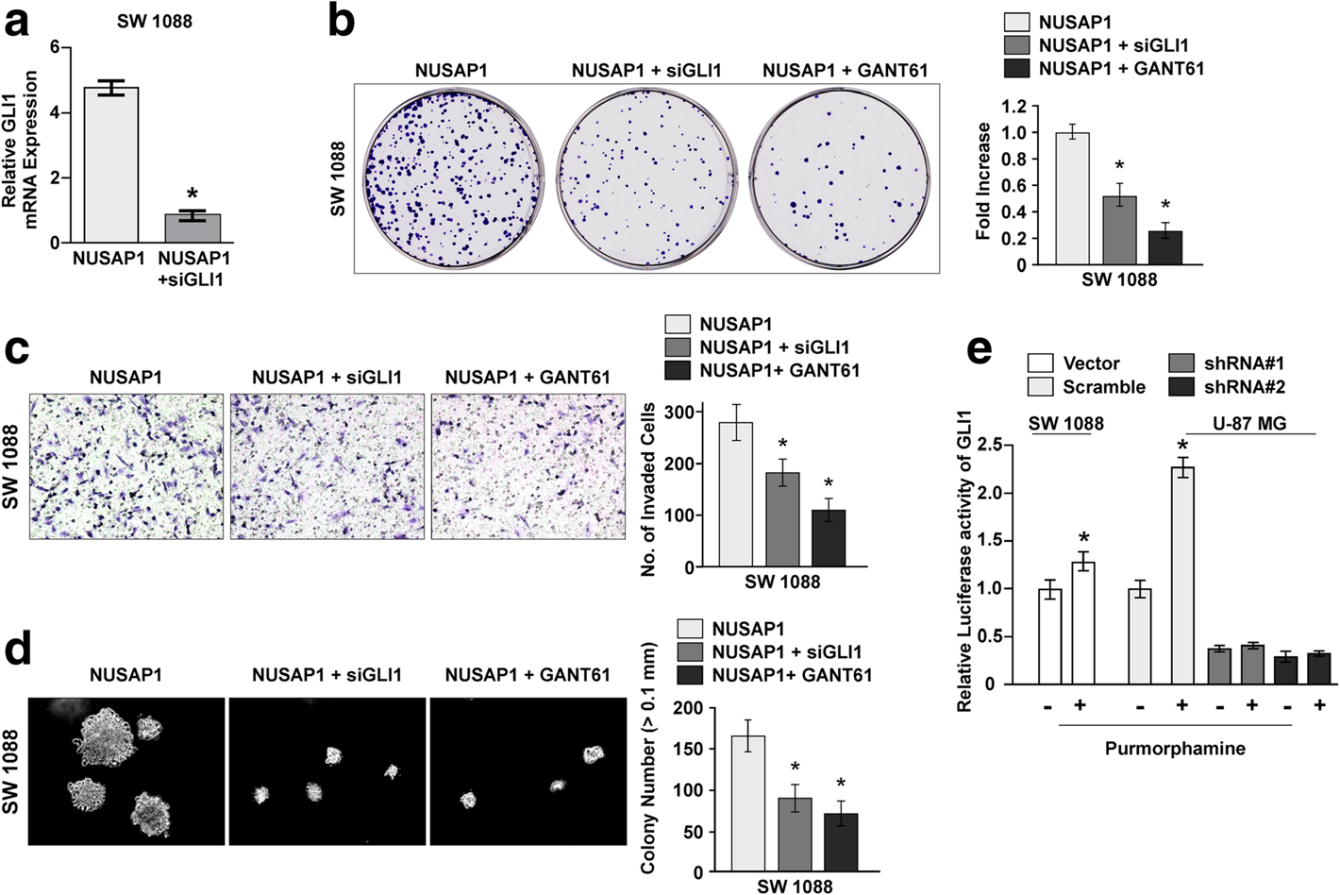
Promotion of tumor aggressiveness by NUSAP1 overexpression via activation of Hedgehog signaling. **a** RT-PCR detection of GLI1 expression in the indicated cells. Error bars represent the mean ± SD values of three independent experiments (*P < 0.05). **b** Representative images (left panel) and quantification (right panel) of the indicated cells in the colony formation assay. Error bars represent the mean ± SD values of three independent experiments (*P < 0.05). **c** Representative images (*left* panel) and quantification (right panel) of the indicated invaded cells analyzed by the Transwell matrix penetration assay. Error bars represent the mean ± SD values of three independent experiments (*P < 0.05). **d** Representative images (*left* panel) and quantification (right panel) of the indicated invaded cells analyzed by the anchorage-independent growth assay. Error bars represent the mean ± SD values of three independent experiments (*P < 0.05). **e** Luciferase reporter assays to determine GLI1 activity in indicated cells when treated with purmorphamine or not. Error bars represent the mean ± SD values of three independent experiments (*P < 0.05)

To confirm whether NUSAP1 promotes tumor aggressiveness by activating the HH pathway, we induced knockdown of the expression of GLI1 by small interfering RNA in NUSAP1-transduced SW1008 cells. As confirmed by RT-PCR, the mRNA level of GLI1 was significantly decreased in NUSAP1-siGLI1 cells (Fig. 6a). Further, the colony formation assay showed that the colony-forming ability of NUSAP1-siGLI1 cells was decreased in comparison with NUSAP1-transduced SW 1088 cells. When the NUSAP1-tranduced SW 1088 cells were treated with GANT61—a small-molecule inhibitor of GLI1- and GLI2-mediated transcription at the nuclear level—the colony formation ability of the cells was decreased (Fig. 6b). Furthermore, NUSAP1-siGLI1 significantly inhibited the cell invasion ability of SW 1088 cells, while treatment of the NUSAP1-transduced cells with GANT61 caused a greater decrease in their cell invasion ability (Fig. 6c). Moreover, NUSAP1-siGLI1 cells showed a decrease in their anchorage-independent growth ability, and treatment of the NUSAP1-transduced cells with GANT61 also caused a significant decrease in their growth ability (Fig. 6d). Moreover, we found that, in response to SMO agonist purmorphamine treatment, the activity of GLI1 was only slightly increased in SW 1088 glioma cells, which displays low NUSAP1 expression, but significantly increased in U-87 MG glioma cells, which exhibits high NUSAP1 expression. Importantly, the simulative effect of SMO agonist purmorphamine on GLI1 activity in U-87 MG glioma cells was dramatically arrogated by NUSAP1 depletion (Fig. 6e). Therefore, these results further supported the notion that NUSAP1 plays critical role in activation of the Hedgehog signaling pathway in glioma cells.

## Discussion

This study revealed that NUSAP1 plays a significant role in promoting aggressiveness in astrocytoma. We found that NUSAP1 expression was significantly upregulated in astrocytoma cell lines and tissues compared with normal astrocytes and brain tissues. We also found that overexpression of NUSAP1 was significantly correlated to poor survival. Moreover, NUSAP1 promoted the invasive ability of astrocytoma cells both under in vitro and in vivo conditions. With regard to the molecular mechanism, we found that upregulation of NUSAP1 promoted the translocation of GLI1 from the cytoplasm to the nucleus and upregulated the downstream genes of the HH pathway in astrocytoma cells. Taken together, our findings provided evidence for the role of NUSAP1 in the progression of astrocytoma and the potential of NUSAP1 as a prognostic biomarker as well as target in astrocytoma treatment.

The involvement of NUSAP1 in cancer has been reported in many studies. For instance, the expression level of NUSAP1 was strongly associated with poor survival in estrogen receptor-positive breast cancer [43]. Further, NUSAP1 could be a biomarker of oral squamous cell carcinoma, as it was reported that downregulation of NUSAP1 suppressed tumor proliferation and enhanced the anti-tumor effect of paclitaxel [44]. Moreover, NUSAP1 promoted prostate cancer progression by increasing the proliferation and invasion of prostate cancer cells [45]. NUSAP1 expression was also found to be upregulated in 95% of human pituitary gonadotroph adenomas [46]. Similar to the findings in benign brain tumor, NUSAP1 was reported to be overexpressed in grade III versus grade I meningiomas [47], and in glioblastoma multiforme [48]. Similar to these findings in various tumors, in this study too, we found that NUSAP1 was dramatically overexpressed in advanced stage astrocytoma patients; moreover, overexpression of NUSAP1 was also predictive of poor overall survival.

There is much evidence to indicate that HH signaling is involved in various cancers, including skin, muscle, esophagus, stomach, pancreas, biliary track, lung, prostate, bladder, oral cavity and brain cancer [49]. HH signaling was found to regulate dorsal brain tumorigenesis, and GLI1 expression was amplified by more than 50-fold in malignant glioma [50, 51]. In agreement with these studies, in our study, we found that GLI1 expression in nucleus was upregulated by NUSAP1. NUSAP1 also upregulated the expression of the downstream targets of HH pathway including PTCH1, HIP1, CCND1, CCNE1 and HDAC1 in astrocytoma. Our findings indicated that NUSAP1 activated HH pathway by promoting GLI1 transport to the nucleus form cytoplasm in astrocytoma cell. GLI1, as a transcription factor, is a vital target gene of HH signaling, and it encodes for the HH signaling interacting protein [52]. However, while GLI1 mRNA expression level was widely known to reflect the activity of the HH signaling pathway [53], GLI1 was also regulated by several protein mediators, such as protein kinase A, glycogen synthase kinase 3β, casein kinase 1α and suppressor of fused [54]. Peterson KA et al. defined a prioritized set of 841 enriched Gli1-binding regions (GBRs) by intersecting ChIP combined with deep sequencing (ChIP-seq) data independently verified in biological replicates [42]. NUSAP1 as one of the genes which have GLI-binding regions, however, has not been validated by any experiments as far as we known. Hence, there are more studies will be needed to testify whether NUSAP1 directly binding to GLI1. Here, we reported that NUSAP1 could promote GLI1 translocation to the cell nucleus, resulting in the activation of HH. However, we could not obtain any direct evidence to precisely show how NUSAP1 promotes GLI1 translocation to the nucleus. Therefore, this is a topic that needs to be studied further in the future.

In conclusion, our study revealed that NUSAP1 plays an important role in astrocytoma progression by promoting the proliferation, invasion and migration of tumor cells. Furthermore, the level of NUSAP1 was notably positively related to poor overall survival in astrocytoma patients. Thus, NUSAP1 might be a potential prognostic biomarker as well as a treatment target in astrocytoma. With regard to the underlying mechanism, NUSAP1 could promote GLI1 translocation to the nucleus and thereby result in the activation of the HH signaling pathway. However, the precise molecular mechanism by which NUSAP1 promotes the nuclear translocation of GLI1 is still unclear and needs to be explored in future studies.

## Conclusion

NUSAP1 contributes to the progression of astrocytoma by enhancing tumor cell invasiveness via activation of the Hedgehog signaling pathway, and that NUSAP1 might be a potential prognostic biomarker as well as a target in astrocytoma.

## Additional files

Additional file 1: Table S1. Clinicopathological characteristics of studied patients and expression of NUSAP1 in 221 glioma specimens. **Table S2.** Correlation between NUSAP1 expression and clinicopathological characteristics of 221 glioma specimens. **Table S3.** Univariate and multivariate analyses of various prognostic parameters in patients with glioma by Cox-regression analysis (DOCX 21 kb)

Additional file 2: Figure S1. NUSAP1 expression is negatively correlated with prognosis in both patients with lower- and higher- grade gliomas. Kaplan–Meier survival curves of patients with lower-grade glioma (grade I-II, *P* < 0.001, left) and with higher-grade glioma (grade III-IV, *P* = 0.018, right). (TIFF 80 kb)

Additional file 3: Figure S2. Upregulation of NUSAP1 promoted the aggressiveness in astrocytoma in vitro. **(A)** Western blotting was used to examine the protein expression of NUSAP1 in A172 cells. α-Tubulin was used as a loading control. **(B)** In the MTT assay, overexpression of NUSAP1 significantly increased the growth rate of the indicated cells, while downregulation of NUSAP1 decreased the growth rate of the indicated cells. Error bars represent the mean ± SD values of three independent experiments. **(C)** Representative images (left panel) and quantification (right panel) of cells in the colony formation assay. Overexpression of NUSAP1 increased, while downregulation of NUSAP1 decreased, the colony-forming ability of the indicated cells. Error bars represent the mean ± SD values of three independent experiments (*P < 0.05). **(D)** Representative images (left panel) and quantification (right panel) of the indicated invaded cells analyzed by the Transwell matrix penetration assay. Error bars represent the mean ± SD values of three independent experiments (*P < 0.05). **(E)** The wound-healing assay was conducted with the indicated cells, and images were taken at 0 and 24 h. Overexpression of NUSAP1 increased, while downregulation of NUSAP1 decreased, the migration ability of the indicated cells. **(F)** Representative images (left panel) and quantification (right panel) of the indicated invaded cells by the anchorage-independent growth assay. Error bars represent the mean ± SD values of three independent experiments (*P < 0.05). **(G)** Western blotting was used to assess the expression of NUSAP1, MMP2, MMP9 and Ki67 in the indicated cells. GAPDH was used as a loading control. (TIFF 13300 kb)

Additional file 4: Figure S3. Upregulation of NUSAP1 activated Hedgehog signaling. (A) Western blotting was used to examine the expression of NUSAP1 and GLI1 in the indicated cells. β-actin and EF1-α were used as loading controls. **(B)** Relative activity of reporter luciferase linked to NUSAP1 and GLI1 in the indicated cells. Error bars represent the mean ± SD values of three independent experiments (*P < 0.05). **(C-D)** RT-PCR detection of PTCH1, HIP1, CCND1, CCNE1 and HDAC1 gene expression in A172 cells. Error bars represent the mean ± SD values of three independent experiments (*P < 0.05). (TIFF 1234 kb)

## Abbreviations

AJCC: American joint committee on cancer
ATCC: American type culture collection
DHH: Desert hedgehog
DMEM: Dulbecco’s modified eagle medium
EF1α: Elongation factor 1 alpha
FBS: fetal bovine serum
GAPDH: Glyceraldehyde-3-phosphate dehydrogenase
GLI1: GLI family zinc finger 1
GSEA: Gene set enrichment analysis
H&E: Hematoxylin-eosin
HH: Hedgehog
IHH: Indian hedgehog
MOD: Mean optical density
MTT: 3-(4,5-dimethylthiazol-2-yl) 2,5-diphenyltetrazolium bromide
NHAs: Primary normal human astrocytes
NUSAP1: Nucleolar and spindle associated protein 1
RFS: Relapse-free survival
SD: Standard deviation
SHH: Sonic hedgehog
SI: Staining index
SMO: Smoothened
WHO: World health organization
