# Supplementary material for: Nucleolar and spindle associated protein 1 promotes the aggressiveness of astrocytoma by activating the Hedgehog signaling pathway
Source: J Exp Clin Cancer Res. 2017 Sep 12;36:127. doi: 10.1186/s13046-017-0597-y (PMC5596921; doi:10.1186/s13046-017-0597-y)
Supplement: Supplementary file 1 — Clinicopathological characteristics of studied patients and expression of NUSAP1 in 221 glioma specimens. Table S2. Correlation between NUSAP1 expression and clinicopathological characteristics of 221 glioma specimens. Table S3. Univariate and multivariate analyses of various prognostic parameters in patients with glioma by Cox-regression analysis (DOCX 21 kb) [file 13046_2017_597_MOESM1_ESM.docx]

**Supplementary Figure Legends**

**Supplementary Figure 1. NUSAP1 expression is negatively correlated with prognosis in both patients with lower- and higher- grade gliomas.** Kaplan–Meier survival curves of patients with lower-grade glioma (grade I-II, *P*<0.001, left) and with higher-grade glioma (grade III-IV, *P*=0.018, right).

**Supplementary Figure 2. Upregulation of NUSAP1 promoted the aggressiveness in astrocytoma in vitro. (A)** Western blotting was used to examine the protein expression of NUSAP1 in A172 cells. α-Tubulin was used as a loading control. **(B)** In the MTT assay, overexpression of NUSAP1 significantly increased the growth rate of the indicated cells, while downregulation of NUSAP1 decreased the growth rate of the indicated cells. Error bars represent the mean ± SD values of three independent experiments. **(C)** Representative images (left panel) and quantification (right panel) of cells in the colony formation assay. Overexpression of NUSAP1 increased, while downregulation of NUSAP1 decreased, the colony-forming ability of the indicated cells. Error bars represent the mean ± SD values of three independent experiments (**P* < 0.05). **(D)** Representative images (left panel) and quantification (right panel) of the indicated invaded cells analyzed by the Transwell matrix penetration assay. Error bars represent the mean ± SD values of three independent experiments (**P* < 0.05). **(E)** The wound-healing assay was conducted with the indicated cells, and images were taken at 0 and 24 h. Overexpression of NUSAP1 increased, while downregulation of NUSAP1 decreased, the migration ability of the indicated cells. **(F)** Representative images (left panel) and quantification (right panel) of the indicated invaded cells by the anchorage-independent growth assay. Error bars represent the mean ± SD values of three independent experiments (**P* < 0.05). **(G)** Western blotting was used to assess the expression of NUSAP1, MMP2, MMP9 and Ki67 in the indicated cells. GAPDH was used as a loading control.

**Supplementary Figure 3. Upregulation of NUSAP1 activated Hedgehog signaling. (A)** Western blotting was used to examine the expression of NUSAP1 and GLI1 in the indicated cells. β-actin and EF1-α were used as loading controls. **(B)** Relative activity of reporter luciferase linked to NUSAP1 and GLI1 in the indicated cells. Error bars represent the mean ± SD values of three independent experiments (**P* < 0.05). **(C-D)** RT-PCR detection of PTCH1, HIP1, CCND1, CCNE1 and HDAC1 gene expression in A172 cells. Error bars represent the mean ± SD values of three independent experiments (**P* < 0.05).

**Supplementary Table S1.** Clinicopathological characteristics of studied patients and expression of NUSAP1 in 221 glioma specimens

| Characteristics | No. of Cases | % |
| --- | --- | --- |
| **Age (years)** |  |  |
| ≤45 | 157 | 71.04 |
| >45 | 64 | 28.96 |
| **Gender** |  |  |
| Male | 145 | 65.61 |
| Female | 76 | 34.39 |
| **WHO grade** |  |  |
| I | 29 | 13.12 |
| II | 72 | 32.58 |
| III | 65 | 29.41 |
| IV | 55 | 24.89 |
| **Vital Status (at follow-up)** |  |  |
| Alive | 95 | 42.99 |
| Dead | 126 | 57.01 |
| **NUSAP1 expression** |  |  |
| Low expression | 69 | 31.22 |
| High expression | 152 | 68.78 |

**Supplementary Table S2.** Correlation between NUSAP1 expression and clinicopathological characteristics of 221 glioma specimens.

| **Characteristics** | | **NUSAP1** | | **Chi-square test**  ***P*-value** |
| --- | --- | --- | --- | --- |
|  |  | **Lower**  **expression** | **Higher expression** |  |
| **Gender** | Male | 46 | 99 | 0.824 |
|  | Female | 23 | 53 |  |
| **Age** | ≤ 45 | 54 | 103 | 0.111 |
|  | > 45 | 15 | 49 |  |
| **WHO grade** | I | 23 | 6 | <0.001 |
|  | II | 36 | 36 |  |
|  | III | 7 | 58 |  |
|  | IV | 3 | 52 |  |
| **Vital Status** | Alive | 49 | 46 | <0.001 |
|  | Death | 20 | 106 |  |

**Supplementary Table S3.** Univariate and multivariate analyses of various prognostic parameters in patients with glioma by Cox-regression analysis

|  | **Univariate analysis** | | | **Multivariate analysis** | | | |
| --- | --- | --- | --- | --- | --- | --- | --- |
|  | **No. patients** | ***P*** | **Regression coefficient (SE)** | ***P*** | **Relative risk** | | **95% confidence interval** |
| **Age** |  | | | | | | |
| **Gender** |  | | | | | | |
| male | 145 | 0.301 | 0.186 | 0.179 | 1.287 | | 0.890-1.860 |
| female | 76 |  |  |  |  |  |  |
| **Age** |  | | | | | | |
| ≤ 45 | 157 | 0.116 | 0.005 | 0.759 | 0.998 | | 0.989-1.008 |
| > 45 | 64 |  |  |  |  |  |  |
| **WHO grade** |  |  |  |  |  | |  |
| **I** | 29 | <0.001 | 0.117 | <0.001 | 2.087 | | 1.612-2.701 |
| **II** | 72 |  |  |  |  |  |  |
| **III** | 65 |  |  |  |  |  |  |
| **IV** | 55 |  |  |  |  |  |  |
| **Expression of NUSAP1** |  | | | | | | |
| **Low** | 69 | <0.001 | 0.279 | <0.001 | | 3.937 | 2.179-7.116 |
| **High** | 152 |  |  |  |  |  |  |
