# Supplementary figures and images for: Nucleolar and spindle associated protein 1 promotes the aggressiveness of astrocytoma by activating the Hedgehog signaling pathway
Source: J Exp Clin Cancer Res. 2017 Sep 12;36:127. doi: 10.1186/s13046-017-0597-y (PMC5596921; doi:10.1186/s13046-017-0597-y)

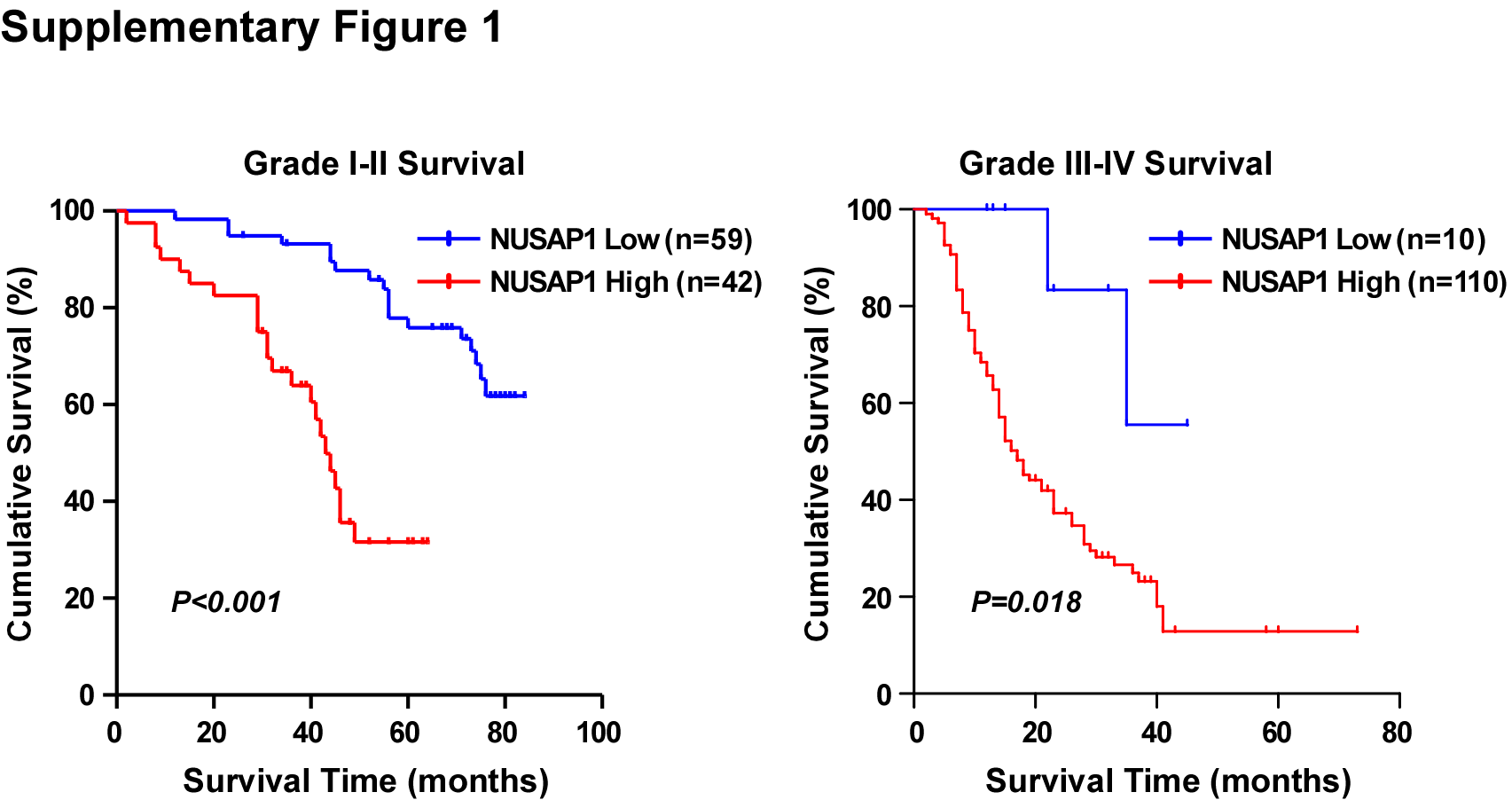

Supplement: Supplementary file 2 — NUSAP1 expression is negatively correlated with prognosis in both patients with lower- and higher- grade gliomas. Kaplan–Meier survival curves of patients with lower-grade glioma (grade I-II, P < 0.001, left) and with higher-grade glioma (grade III-IV, P = 0.018, right). (TIFF 80 kb) [file 13046_2017_597_MOESM2_ESM.tif]

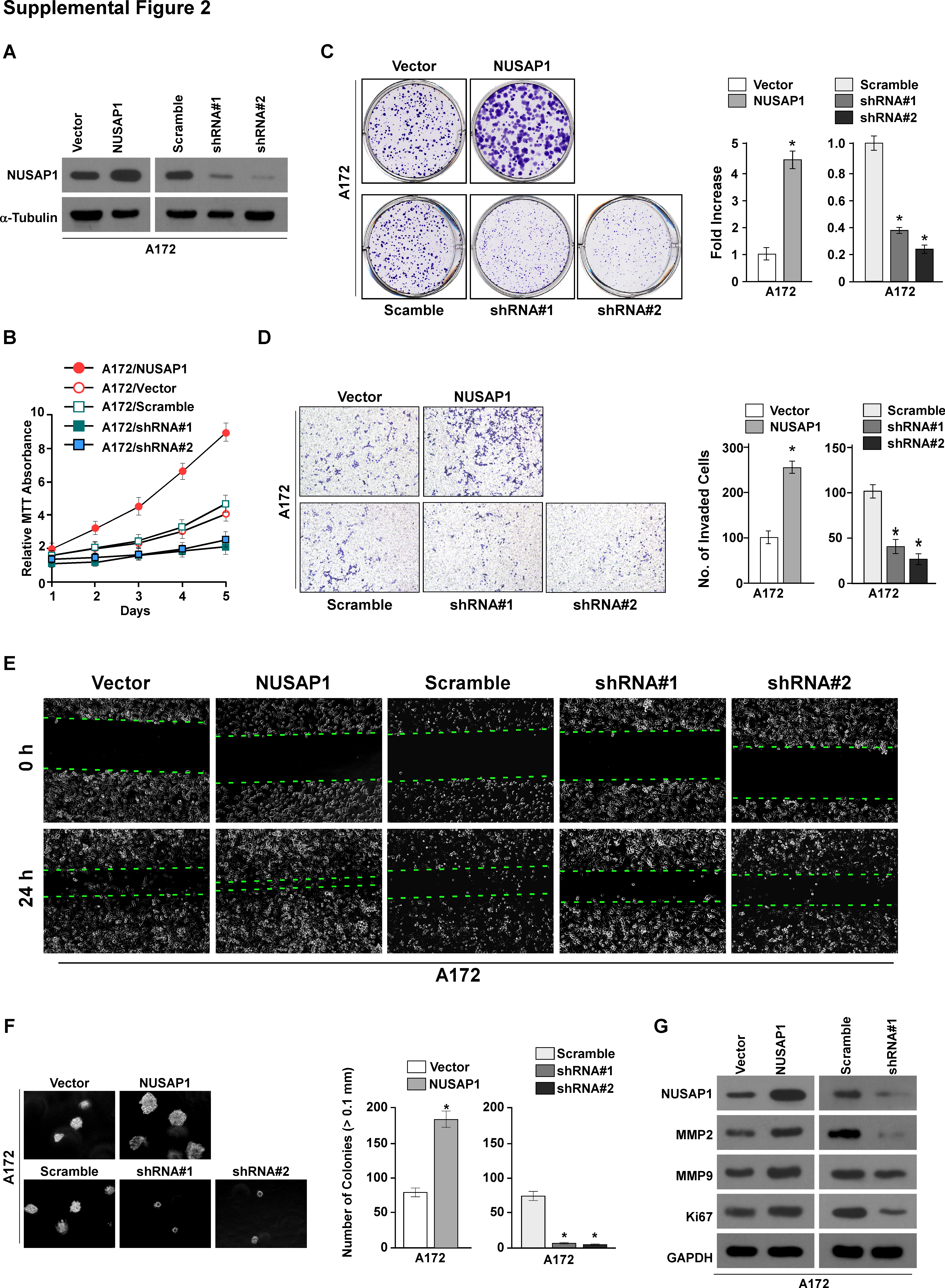

Supplement: Supplementary file 3 — Upregulation of NUSAP1 promoted the aggressiveness in astrocytoma in vitro. (A) Western blotting was used to examine the protein expression of NUSAP1 in A172 cells. α-Tubulin was used as a loading control. (B) In the MTT assay, overexpression of NUSAP1 significantly increased the growth rate of the indicated cells, while downregulation of NUSAP1 decreased the growth rate of the indicated cells. Error bars represent the mean ± SD values of three independent experiments. (C) Representative images (left panel) and quantification (right panel) of cells in the colony formation assay. Overexpression of NUSAP1 increased, while downregulation of NUSAP1 decreased, the colony-forming ability of the indicated cells. Error bars represent the mean ± SD values of three independent experiments (*P < 0.05). (D) Representative images (left panel) and quantification (right panel) of the indicated invaded cells analyzed by the Transwell matrix penetration assay. Error bars represent the mean ± SD values of three independent experiments (*P < 0.05). (E) The wound-healing assay was conducted with the indicated cells, and images were taken at 0 and 24 h. Overexpression of NUSAP1 increased, while downregulation of NUSAP1 decreased, the migration ability of the indicated cells. (F) Representative images (left panel) and quantification (right panel) of the indicated invaded cells by the anchorage-independent growth assay. Error bars represent the mean ± SD values of three independent experiments (*P < 0.05). (G) Western blotting was used to assess the expression of NUSAP1, MMP2, MMP9 and Ki67 in the indicated cells. GAPDH was used as a loading control. (TIFF 13300 kb) [file 13046_2017_597_MOESM3_ESM.tif]

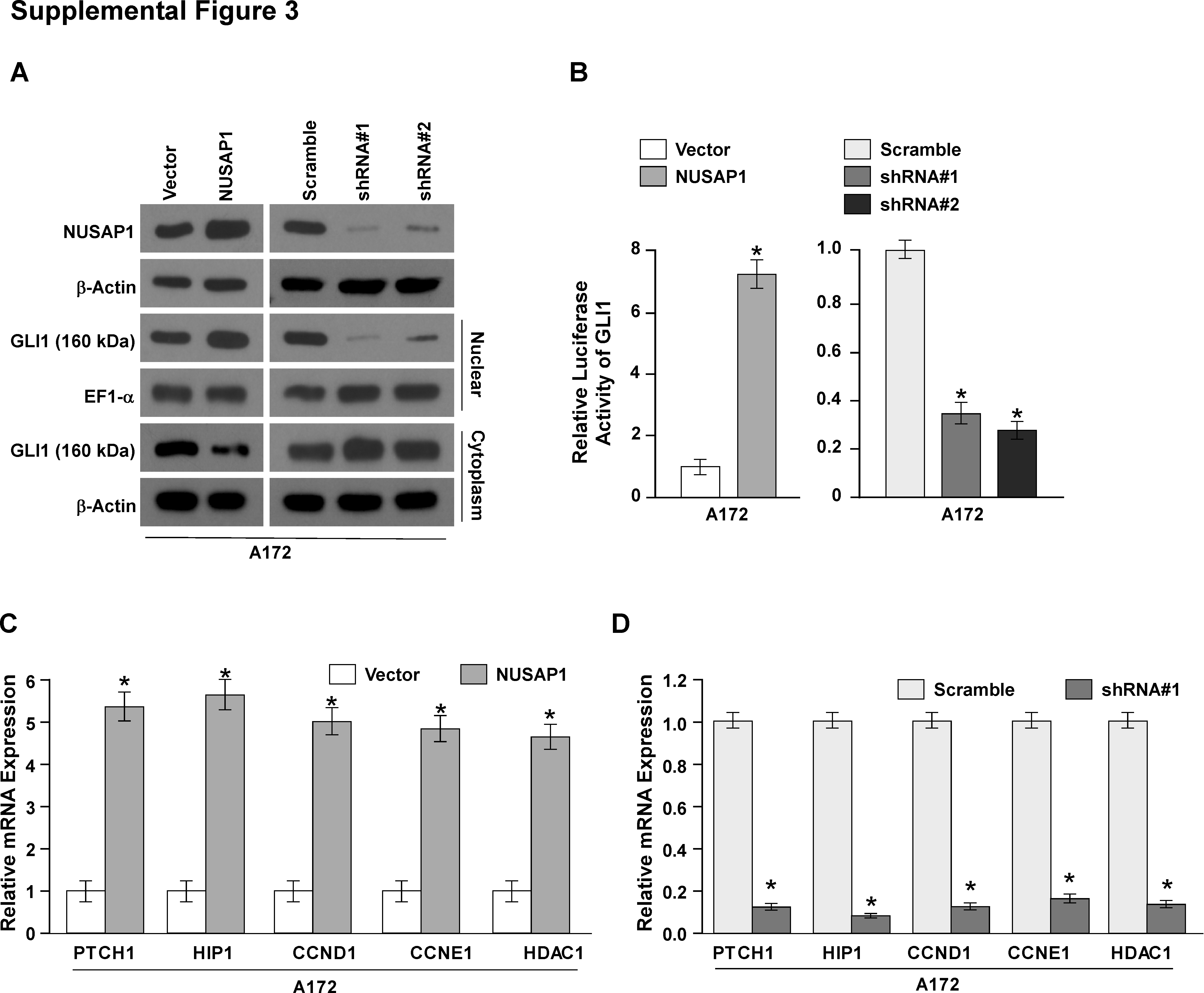

Supplement: Supplementary file 4 — Upregulation of NUSAP1 activated Hedgehog signaling. (A) Western blotting was used to examine the expression of NUSAP1 and GLI1 in the indicated cells. β-actin and EF1-α were used as loading controls. (B) Relative activity of reporter luciferase linked to NUSAP1 and GLI1 in the indicated cells. Error bars represent the mean ± SD values of three independent experiments (*P < 0.05). (C-D) RT-PCR detection of PTCH1, HIP1, CCND1, CCNE1 and HDAC1 gene expression in A172 cells. Error bars represent the mean ± SD values of three independent experiments (*P < 0.05). (TIFF 1234 kb) [file 13046_2017_597_MOESM4_ESM.tif]
